# Updating sentences lists for assessment speech perception

**DOI:** 10.1590/2317-1782/20202020301

**Published:** 2022-01-10

**Authors:** Maria Madalena Canina Pinheiro, Michele Gindri Vieira, Lara Malafaia Vieira, Isadora Koerich, Isadora Rosseto, Cristiane Lazzarotto-Volcão, Stephan Paul

**Affiliations:** 1 Departamento de Fonoaudiologia, Universidade Federal de Santa Catarina – UFSC - Florianópolis (SC), Brasil.; 2 Centro Catarinense de Reabilitação/Centro Especializado em Reabilitação, Secretaria de Estado da Saúde de Santa Catarina- Florianópolis (SC), Brasil.; 3 Curso de Fonoaudiologia, Universidade Federal de Santa Catarina – UFSC - Florianópolis (SC), Brasil.; 4 Departamento de Língua e Literatura Vernáculas, Universidade Federal de Santa Catarina – UFSC - Florianópolis (SC), Brasil.; 5 Departamento de Engenharia Mecânica, Universidade Federal de Santa Catarina – UFSC - Florianópolis (SC), Brasil.

**Keywords:** Speech Perception, Auditory Perception, Hearing, Speech Discrimination Tests, Hearing Tests

## Abstract

**Purpose:**

Adapt a list of sentences for a speech intelligibility test.

**Methods:**

A speech material data base consisting of 200 phonetically balanced sentences was analyzed and partially updated. In the first stage, 60 reviewers, specialists in linguistics and speech and hearing science, analyzed the sentences in relation to the parameters of familiarity, meaning and predictability using an on-line questionnaire. Cronbach's Alpha coefficient was used to analyze the internal consistency of the questionnaire. In the second stage, the reviewers analyzed whether they were in accordance with the criteria indicated by the literature for the construction of sentences.

**Results:**

In the first stage, the responses of 15 reviewers who completed the entire questionnaire were analyzed. Agreement between reviewers was high for all criteria. 71 sentences were recommended for modification in the first stage, with predictability being the most indicated parameter as requiring change. In the second stage, 28 more sentences were selected for adjustment, with the presence of a proper name in the sentence being the most frequently cited criterion.

**Conclusion:**

It was possible to adapt a list of sentences in order to provide speech language therapists with a free of charge speech perception protocol. It is hoped that this new test can assist in standardizing assessment for normal hearing adults and individuals with hearing loss in Brazilian Portuguese.

## INTRODUCTION

The ability to understand is fundamental for social integration since it is the first step that enables communication between individuals. It is considered the most important aspect to be evaluated in auditory functioning, generating data that show how individuals listen and understand during their daily routine^(1)^.

Collaboration between Speech-Language Therapy and Linguistics has developed techniques for early diagnosis of alterations in individuals’ language and for the development of protocols to assess speech comprehension. The field of speech processing in engineering is undergoing constant evolution, contributing technological resources that improve, standardize and automate the testing used in the audiological area^(2)^. There have been significant advances in the assessment of speech perception owing to this relationship^(3)^.

In the literature, speech materials that have already been developed to evaluate the detection and discrimination of sounds and words were observed^(4,5)^, in addition to the ability to recognize monosyllables, words, and sentences^(6-8)^.

To assess real communication, the literature recommends the application of sentences with competitive noise^(8-11)^. These sentences can be used both with normal listeners^(12)^ and individuals with hearing loss^(6)^. However, its most appropriate application is for evaluating and monitoring candidates for and users of hearing aids (HA) and cochlear implants (CI)^(13,14)^.

The analysis of the national literature showed that currently, there are few options for speech perception tests available for use in the assessment of adults in clinical practice. In this context, a well-known test is the List of Sentences in Portuguese^(6)^, which is available on Compact Disc. This test requires the speech-language therapist to adjust speech and noise levels. Several studies use this material and recognize its wide applicability^(13,15,16)^.

The Sentence Lists of the Audiological Research Center (*Centro de Pesquisas Audiológicas - CPA*) is another test to assess speech recognition that uses phonetically balanced sentences and is widely used in most CI services. However, it is used in most hands-free services, and there is no uniformity in its application^(7)^.

The Hearing in Noise Test (HINT) is widely used, especially in the research field^(8)^. Its advantage is that of being internationally recognized, while the high cost of its license for use in the clinical context is a disadvantage^(17)^.

To create a new speech recognition test for speech-language therapy, we analyzed speech banks of Brazilian Portuguese (BP). Speech banks are files composed of a large number of sentences that make it possible to capture variations and changes in a speech community^(18,19)^.

Alcaim^(12)^ developed a well-known speech bank in Brazil and it was adapted by Seara^(20)^. This bank consists of phonetically balanced sentences, which contain 35 segments (phonemes and their variations) of BP with a total occurrence of 20,178 sounds throughout the sentences.

The research carried out by Seara had academic purposes and stood out for its rigor in terms of phonetic balance, which was more complete than the author who proposed them^(12)^. However, given the clinical purposes of this study and the concern to adjust the vocabulary, meaning, and predictability of the speech material, we needed to update the sentences.

To make available a test that assesses speech recognition and facilitates the performance of the speech-language therapist for clinical practice, this study had the general objective of adapting a list of sentences to assess speech recognition in adults.

## METHODS

The present study was observational, cross-sectional and analytical, and took place from June to August 2019. It was approved by the Research Ethics Committee of the Federal University of Santa Catarina (UFSC) and has the approval number of the Ordinance 1,997,931 and CAAE56838816.7.00000.0121.

In the present paper, we sought to adapt lists of sentences to assess speech recognition, based on a speech bank with phonetically balanced sentences, adapted by Seara^(20)^ and authored by Alcaim^(12)^. Seara's speech bank^(20)^ is composed of 200 sentences distributed equally into 20 lists with 10 sentences each. These sentences are widely used as a speech bank in speech intelligibility tests with normal listeners in the Engineering area.

This study was carried out in three stages: analysis by reviewers through an online questionnaire, updating of sentences according to construction criteria based on the literature, and a pilot study with normally hearing individuals.

### 1^st^ stage - Analysis by the reviewers through an online questionnaire

At this stage, we built an online questionnaire for the reviewers to analyze Seara's sentences^(20)^.

The speech bank sentences were transferred to an online platform (SurveyMonkey), aiming to facilitate the access and participation of reviewers from various regions of the country.

We invited two groups of reviewers made up of linguists and speech-language therapists with experience in auditory rehabilitation to carry out the sentence analysis. Recruited linguists needed to have at least a master's degree and speech-language therapists needed to have at least three years-experience. These professionals were appointed by professors and professionals with experience in the areas of phonetics, phonology, and auditory rehabilitation. Before submitting the questionnaire online, the reviewers were contacted by email or social media.

The group of linguists was called GL and was divided into two groups (GL1 and GL2). The group of speech-language therapists was called GR, and was divided into GR1 and GR2.

Due to a large number of sentences, we decided to divide 187 sentences into four subgroups to facilitate analysis, with 47 sentences being allocated for subgroups GL1, GL2, and GR2 and 46 for subgroup GR1. In addition to these different sentences, each subgroup also analyzed 13 randomly chosen, common sentences to evaluate the agreement of all subgroups.

Prior to sentence analysis, reviewers needed to answer questions about their undergraduate and graduate education, their institutional affiliation, whether they had experience in acoustic analysis, and what their field of study was. Therapists were asked about the year they graduated in Speech-Language Pathology, the time spent working in speech-language therapy, and whether speech recognition tests were applied.

For the sentence analysis, each reviewer was given a classification of marks for the following criteria: familiarity, sentence meaning, and predictability. We collected the answers using solid-line scales, collated in SurveyMonkey through a slider bar ranging from zero (0) to one hundred (100).

The classification of the familiarity of each sentence was realized according to how common or well-known the sentences were to the reviewer. In this item, 0 represented a sentence uncommon or unknown for the reviewer, while grade 100, was very common or well-known.

Sentence meaning was evaluated according to the meaning mobilized by the sentence. In this item, zero represents an absence of meaning, while 100, full meaning.

Predictability was classified according to the expectation of a continuation of the sentence from some initial words. 0 represents no expectation regarding what could complement the sentence while 100indicates an ability to predict the end by reading only the beginning of the sentence, that is, significant predictability.

At the end of the first stage, the data were descriptively analyzed and, to verify the internal consistency of the answers to the questionnaire applied to the reviewers, we used Cronbach's Alpha test, calculated using SPSS software. Cronbach's Alpha coefficient measures the relationship between the answers of a questionnaire, through an analysis of the research participants' answers. This coefficient reflects the degree of covariance between the items on a scale, with a lower sum of the items' variance, indicating a more consistent instrument. Values greater than or equal to 0.70 are indicative of adequate internal consistency^(21)^.

### 2^nd^ stage - Updating the sentences according to the construction criteria based on the literature

In the second stage of the research, three reviewers who did not participate in the first analysis updated the sentences indicated by the reviewers from the first stage, carrying out the recommended modifications for the criteria of familiarity, sentence meaning, and predictability. The reviewers of the second stage were two doctors of linguistics and one doctor of auditory rehabilitation.

The second stage reviewers also verified whether they were following the construction parameters of sentences based on the literature^(6,22,23)^: exclusion of proper names; affirmative sentences with simple and compound period; sentences consisting of three to seven phonological words, and with a low level of abstraction. In this second stage, the modifications in each sentence also prioritized the semantic aspect over the phonetic one, to adapt the sentences to the study objectives.

We considered a phonological word a phonological or prosodic word that only has one primary accent (tonic). This criterion was used to facilitate counting the number of correct answers when applying the speech recognition test.

After these adjustments, we organized the sentences into 10 lists with 20 sentences each to facilitate the clinical assessment of speech recognition. In each of the 20-sentence lists, the number of sentences modified by the reviewers and those considered original (about Seara's work) was also analyzed to achieve a more uniform distribution in each list, to maintain the phonetic balance of each list as much as possible.

We needed to adjust the number of phonological words per list to obtain 100 phonological words in each of the 10 lists. For this, we reviewed the lists and adjusted the sentences that had already been indicated for modification by the first stage reviewers. In this way, the original sentences were not altered.

At the end of the review, each list presented 100 phonological words, with each one in the list corresponding to 1%, that is, the number of phonological word errors obtained by the patient in a given list will be reduced from the total score (100%) with the result being expressed as a percentage.

### 3^rd^stage - Pilot study with normal hearing subjects

To assess the clinical applicability of the sentence lists, we carried out a pilot study with three young (mean age 23.33 years), normal-hearing individuals, without attention and/or memory impairments, in the Department's Laboratory of Vibration and Acoustics of The Mechanical Engineering Department at the UFSC. During the application of the lists, the individual remained with a circum-aural SennheiserHDA200 headset in an acoustic booth, and we used the Inter-acoustics model AC 40 audiometer. The 200 sentences were presented live, at a fixed intensity of 50 dBNA bilaterally, controlled by the VU meter. The same evaluator applied all the lists to the individuals on the same day, taking breaks to avoid fatigue.

Data analysis from the second and third stages was descriptive.

## RESULTS

The sentences of this study were sent in the first stage through an online questionnaire to 60 reviewers, including 37 linguists, and 23 therapists. In the group of linguists (GL), 26 reviewers accessed the questionnaire, but only seven (18.91%) analyzed all the items therein. In the therapist group (GR), 21 reviewers accessed the questionnaire, but only eight (34.78%) analyzed all the items therein. The final sample generated the complete evaluations of 15 reviewers, including seven linguists and eight therapists.

For the initial training of the seven individuals in the GL, we observed that the majority were speech-language therapists (42.85%), followed by Portuguese-Spanish Graduates (28.59%), English Graduates (14.28%), and Electrical Engineering graduates (14.28%). Of these participants, most had a postgraduate degree in Linguistics (42.85%).

In the GR, the eight participants (100%) were speech-language therapists with an average of 19 and a half years of study and 17 and a half years of experience in the area of auditory rehabilitation. Regarding their experience in applying speech perception tests, six (75%) responded that they applied speech recognition tests.

GL participants informed, in the questionnaire, about their experience in previous acoustic analysis, with six (85.70%) reviewers having experience in acoustic analysis and one (14.30%) not responding to the question.

Table 1 shows the descriptive statistics of the classifications of the 13 sentences in common analyzed by the GL and GR groups.

**Table 1:** Descriptive analysis of the judges' answers for the criteria familiarity, sentence meaning and predictability in common sentences

		Mean	Median	Standard Deviation	Maximum	Minimum
Familiarity	GL	86.10%	90.37	10.94	100.00	0.00
	GR	84.38%	83.75	10.12	100.00	0.00
	Total	85.24%	87.50	10.47	100.00	0.00
Sentence Meaning	GL	90.39%	92.50	6.68	100.00	35.00
	GR	90.01%	89.83	7.11	100.00	25.00
	Total	90.20%	90.75	6.83	100.00	25.00
Predictability	GL	48.21%	45.75	16.13	100.00	0.00
	GR	65.31%	64.54	18.76	100.00	0.00
	Total	56.76%	54.87	19.35	100.00	0.00

Caption: GL = Linguists Group; GR = Rehabilitation Group

The predictability criterion is considered the inverse of familiarity and sentence meaning. For this criterion, a score of zero represents low predictability, while in the familiarity and sentence meaning items, the maximum score of 100 represented significant familiarity or a fully meaningful sentence.

In Table 1, we observed that the two groups produced similar responses for the familiarity and sentence meaning criteria and that the therapist group evaluated the sentences as more predictable than the linguists. Cronbach's Alpha coefficient showed that for the analysis of the 13 common sentences, the agreement of the reviewers for the parameters of predictability (0.986), familiarity, and sentence meaning (0.960) was high.

Regarding the analysis of the 187 sentences, evaluated by the subgroups, Cronbach's Alpha coefficient showed that the agreement found in the predictability criteria was 0.99 for the linguist group and 0.97 for the therapist group. The familiarity and sentence meaning parameters were 0.93 for the linguist group and 0.92 for the therapist group.

We carried out the analysis of the subgroups separately for the reviewers’ answers to the online questionnaire. Table 2 shows the descriptive analysis of the evaluations for the 47 sentences considered by each subgroup against each criterion.

**Table 2:** Descriptive analysis of the judges' responses by subgroups for the criteria familiarity, sentence meaning and predictability of different sentences

		Mean	Median	Standard Deviation	Maximum	Minimum
Familiarity	GL1	90.10%	95.00	11.56	100.00	10.00
	GL2	80.43%	80.25	12.47	100.00	7.00
	GR1	80.57%	83.33	15.92	100.00	0.00
	GR2	82.15%	83.33	10.96	100.00	18.00
	Total	83.33%	86.66	13.36	100.00	0.00
Sentence Meaning	GL1	87.53%	93.50	13.10	100.00	20.00
	GL2	87.62%	89.50	9.97	100.00	11.00
	GR1	85.49%	87.12	13.33	100.00	0.00
	GR2	86.25%	89.00	12.12	100.00	23.00
	Total	86.73%	89.50	12.13	100.00	0.00
Predictability	GL1	29.65%	31.25	13.90	100.00	0.00
	GL2	55.95%	55.50	12.67	100.00	0.00
	GR1	47.84%	47.87	12.43	100.00	0.00
	GR2	76.77%	78.00	13.28	100.00	17.00
	Total	52.58%	53.50	21.39	100.00	0.00

Caption: GL1 = Linguists Group 1; GL2 = Linguists Group 2; GR1 = Rehabilitation Group 1; GR2 = Rehabilitation Group 2

In Table 2, we observed that the GL1 subgroup attributed higher scores to the sentences for familiarity. In the other subgroups, the evaluation average was similar. Also, the GL1 considered the sentences to be less predictable, while GR2 considered the sentences more predictable. There was greater divergence in the subgroup answers for the evaluation of predictability, in comparison with the familiarity and sentence meaning criteria.

From these analyses, of the 187 different sentences, we decided to modify those that had an evaluation below 69.97% for the familiarity criteria (Average 83.33 - SD 13.36), below 74.60% for sentence meaning (Mean 86.73 - SD 12.13) and 73.97% or above (Mean 52.58% + SD 21.39) for predictability.

The percentage threshold that verified the need to modify the 13 sentences analyzed by all subgroups was calculated separately. We chose to modify those sentences with an evaluation below 74.77% for familiarity (Average 85.24 - SD 10.47), below 83.37% for sentence meaning (Average 90.20 - SD 06.83), and76.12% or above for (Average 56.76% + SD 19.35) for predictability.

**Figure 1:**
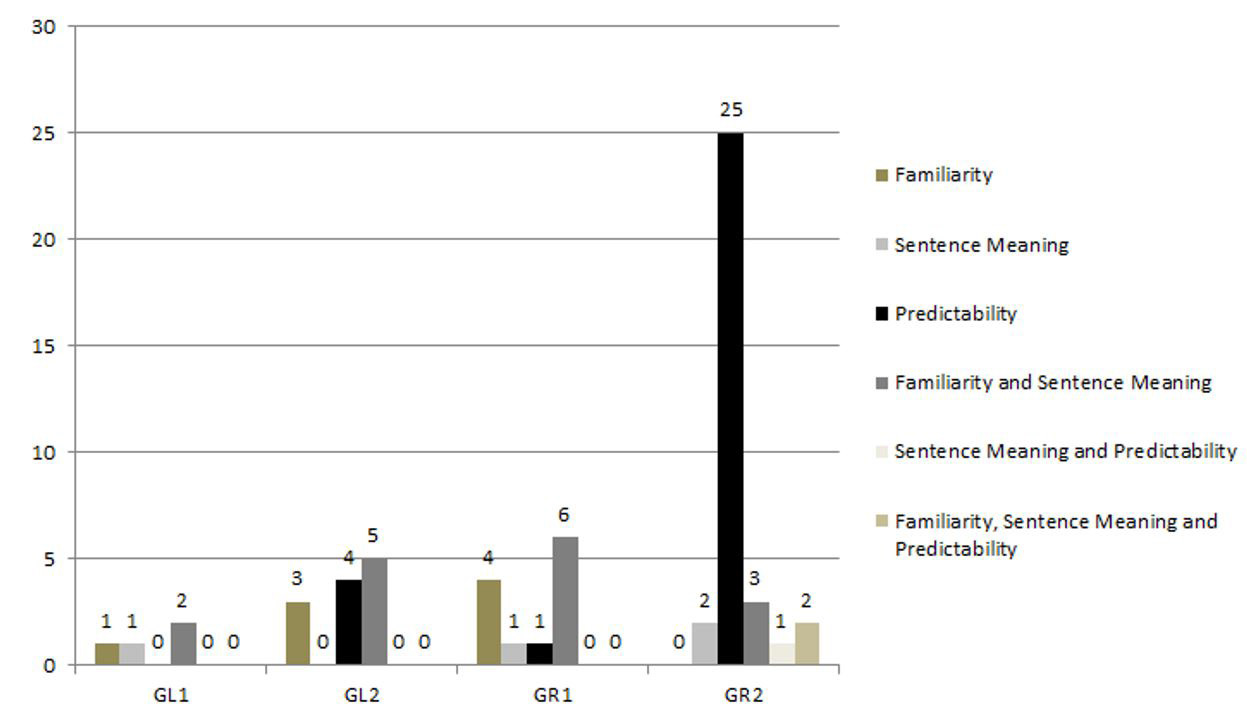
Distribution of the number of different sentences to be modified by each subgroup of judges according to the evaluated criteria

**Figure 2:**
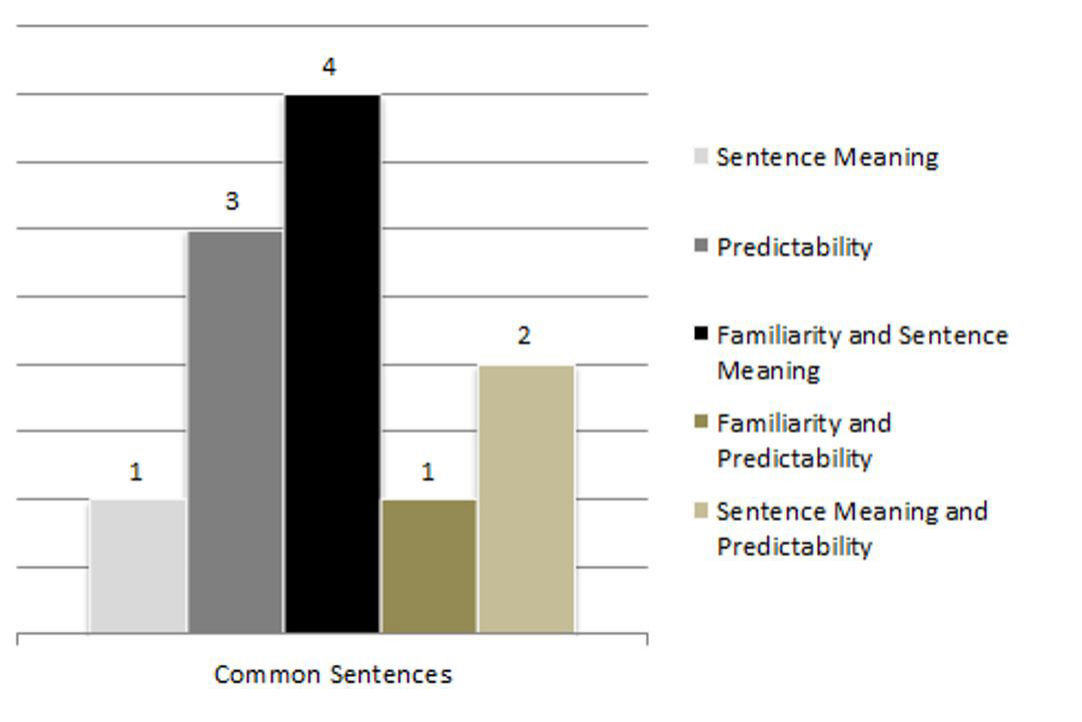
Distribution of the number of sentences in common to be modified according to the evaluated criteria

Figures 1 and 2 illustrate the number of sentences judged by the pre-established criteria of predictability, familiarity, sentence meaning, and concomitant criteria, which will need to be modified based on the criteria described above, with 1 referring to different sentences and 2 to common sentences. Figure 1 shows the analysis of the number of sentences evaluated by each subgroup of reviewers.

Caption: GL1 = Linguists Group 1; GL2 = Linguists Group 2; GR1 = Rehabilitation Group 1; GR2 = Rehabilitation Group 2

Figure 1 shows that GR2 was the subgroup that most indicated sentences for modification based on the predictability criterion while GR1 did so based on the familiarity and sentence meaning criteria.

Of the 187 different sentences analyzed, 60 (32.00%) were modified. Of these, eight (13.33%) were modified according to the familiarity criterion, three (5.0%) according to the sentence meaning criterion, 30 (50.00%) according to the predictability criterion, 16 (26.66%) according to the familiarity and sentence meaning criteria concomitantly, one (1.66%) according to the sentence meaning and predictability criteria, and two (3.33%) according to the three criteria concomitantly.

Eleven (84.6%) of the 13 sentences analyzed by all subgroups were evaluated in relation to the criteria as requiring modification, one (9.09%) according to the sentence meaning criterion, three (27.27%) according to the predictability criterion, four (36.36%) according to the familiarity and sentence meaning criteria concomitantly, one (9.09%) according to the familiarity and predictability criteria concomitantly, and two (18.18%) according to the sentence meaning and predictability criteria concomitantly.

In the second stage, the reviewers identified 28 more sentences to modify according to parameters indicated in the literature, of which three were non-affirmative sentences, five presented excessive abstraction or inadequate semantics, seven contained more than eight phonological words and 13 contained proper names.

The number of phonological words per list was also analyzed at this stage. Only list 7 had 100 phonological words. Lists 1 and 5 exceeded the recommended number of phonological words per list. Lists 2, 3, 4, 6, and 8 to 10 ranged from 89 to 98 words per list. The standardization of the number of phonological words per list to 100 was carried out in the sentences that the first stage reviewers recommended for modification.

Chart 1 shows the complete list with the final 200 sentences.

**Chart 1:** Sentence Recognition Test Lists

List 1								List 2							
1	Recebi	meu	pai	para	almoçar.			1	Esse	tema	foi	falado	na conferência.		
	1	2	3	4	5				1	2	3	4	5		
2	Minha	sobrinha	tem	um lindo	casaco.			2	Queremos	discutir	o orçamento.				
	6	7	8	9	10				6	7	8				
3	Os pesquisadores	não	acreditam	nessa	história.			3	Meu	neto	corre	bem	rápido	na rua.	
	11	12	13	14	15				9	10	11	12	13	14	
4	O trabalho	pode	mudar	a vida.				4	Ela	seguia	discretamente.				
	16	17	18	19					15	16	17				
5	Foi	muito	difícil	entender	a canção	de natal.		5	O sorvete	do menino	tem	cobertura	de caramelo.		
	20	21	22	23	24	25			18	19	20	21	22		
6	Nós	nascemos	aqui	neste	hospital.			6	A pesca	é	proibida	nesse	canto.		
	26	27	28	29	30				23	24	25	26	27		
7	A sujeira	lançada	no rio	contamina	os peixes.			7	Aqui	é	um ótimo	lugar	para	tomar	chá.
	31	32	33	34	35				28	29	30	31	32	33	34
8	Meu	voo	foi	marcado	para	às cinco	horas.	8	É hora	do homem	se humanizar	mais.			
	36	37	38	39	40	41	42		35	36	37	38			
9	O candidato	falou	como	se já	estivesse	eleito.		9	O subúrbio	da cidade	tem	muita	história.		
	43	44	45	46	47	48			39	40	41	42	43		
10	Seu	amigo	chegou	na frente	do mercado.			10	A feira	livre	não	funcionará	amanhã.		
	49	50	51	52	53				44	45	46	47	48		
11	O prêmio	será	entregue	na sessão	solene.			11	Cada	sociedade	tem	uma	cultura	única.	
	54	55	56	57	58				49	50	51	52	53	54	
12	A flor	mais	fascinante	fica	aqui.			12	Nosso	filho	ama	os animais.			
	59	60	61	62	63				55	56	57	58			
13	Em muitas	cidades	a população	está	diminuindo.			13	A loja	de produtos	naturais	fica	na próxima	rua.	
	64	65	66	67	68				59	60	61	62	63	64	
14	A inauguração	da praça	é	quarta-	feira.			14	Essa	medida	foi	devidamente	alterada.		
	69	70	71	72	73				65	66	67	68	69		
15	Não	é	permitido	fumar	no interior	do ônibus.		15	A torcida	marcou	a temporada	de jogos.			
	74	75	76	77	78	79			70	71	72	73			
16	O tigre	entrou	no combate.					16	Receba	minha	prima	na festa.			
	80	81	82						74	75	76	77			
17	A cantora	foi	apresentar	um grande	sucesso.			17	Quero	te ver	bem	feliz	quando	ele	voltar.
	83	84	85	86	87				78	79	80	81	82	83	84
18	Eu	viajarei	para	bem	longe	amanhã.		18	A principal	personagem	no filme	é	uma	gueixa.	
	88	89	90	91	92	93			85	86	87	88	89	90	
19	A bolsa	de valores	está	em alta.				19	Seu	crime	foi	ignorado	pela	vítima.	
	94	95	96	97					91	92	93	94	95	96	
20	Perguntei	o horário	com gentileza.					20	A casa	é	enfeitada	com rosas.			
	98	99	100						97	98	99	100			
List 3								List 4							
1	A casa	foi	vendida	no leilão.				1	Ela	não	tem	fome	quando	sai	de casa.
	1	2	3	4					1	2	3	4	5	6	7
2	Nosso	telefone	está	mudo.				2	A união	dos alunos	rendeu	mudanças.			
	5	6	7	8					8	9	10	11			
3	Dei	dinheiro	para	o menino	comprar	um jogo.		3	Depois	do almoço	te encontro	pro	chá.		
	9	10	11	12	13	14			12	13	14	15	16		
4	Ainda	faltam	seis	minutos.				4	A secretária	não	quer	me dizer	a data.		
	15	16	17	18					17	18	19	20	21		
5	O enfeite	da porta	era	feito	de palha.			5	Faz	um desvio	em direção	ao mar.			
	19	20	21	22	23				22	23	24	25			
6	É	a minha	chance	de esclarecer	a notícia.			6	A pequena	nave	pousou	num	campo	de guerra.	
	24	25	26	27	28				26	27	28	29	30	31	
7	A senhora	sempre	colhe	frutas	maduras.			7	O adiantamento	surpreendeu	a mim	e a todos.			
	29	30	31	32	33				32	33	34	35			
8	Usar	mais	aditivo	pode	ser	desastroso.		8	É	bom	ver	o menino	colhendo	flores.	
	34	35	36	37	38	39			36	37	38	39	40	41	
9	É	fundamental	encontrar	a razão	da existência	humana.		9	Ela	ficou	na fazenda	por uma	hora.		
	40	41	42	43	44	45			42	43	44	45	46		
10	A ideia	é	falha,	mas	interessante.			10	Meu	pai	viajará	de avião	amanhã.		
	46	47	48	49	50				47	48	49	50	51	
11	Um casal	de amigos	saiu	para	festejar.			11	O jardim	exige	muito	trabalho.			
	51	52	53	54	55				52	53	54	55			
12	A saída	para	a crise	dele	é	o diálogo.		12	Eu	venho	jantar	em casa	na quinta-	feira.	
	56	57	58	59	60	61			56	57	58	59	60	61	
13	Esse	filme	parece	bastante	divertido.			13	Hoje	eu	acordei	calmo.			
	62	63	64	65	66				62	63	64	65			
14	Será	bom	que ele	estude	o assunto.			14	Foi	um prazer	conhecer	sua	amiga.		
	67	68	69	70	71				66	67	68	69	70		
15	A garota	foi	no mercado	à noite.				15	A correção	do exame	foi	coerente.			
	72	73	74	75					71	72	73	74			
16	Defender	a ecologia	é	manter	a vida.			16	Fiz	a reserva	para	nosso	passeio	ecológico.	
	76	77	78	79	80				75	76	77	78	79	80	
17	Ele	não	entende	quando	falam	rapidamente.		17	Minha	filha	é	especialista	em música	sacra.	
	81	82	83	84	85	86			81	82	83	84	85	86	
18	A duração	do simpósio	é	de cinco	dias.			18	Os quadros	da parede	mostram	a natureza.			
	87	88	89	90	91				87	88	89	90			
19	O discurso	de abertura	foi	muito	vaiado.			19	Eu	precisei	de tempo	na conferência.			
	92	93	94	95	96				91	92	93	94			
20	A lojinha	não	fica	na esquina.				20	Os hotéis	do litoral	são	fantásticos	para	descansar.	
	97	98	99	100					95	96	97	98	99	100	
List 5								List 6							
1	O analfabetismo	é	um problema	grave.				1	Ele	caminhou	na praia	calma.			
	1	2	3	4					1	2	3	4			
2	Sei	que amanhã	atingiremos	a meta	proposta.			2	Tenho	muito	orgulho	da nossa	gente.		
	5	6	7	8	9				5	6	7	8	9		
3	Eu	vi	uma	blusa	na loja.			3	Esse	projeto	terá	grande	sucesso.		
	10	11	12	13	14				10	11	12	13	14		
4	Uma	índia	andava	na floresta.				4	Reflita	antes	e discuta	depois.			
	15	16	17	18					15	16	17	18			
5	Meu	irmão	tem	motivos	para	comemorar.		5	Eu	tirei	o título	de eleitor	neste	mês.	
	19	20	21	22	23	24			19	20	21	22	23	24	
6	A corrida	de inverno	foi	uma	alegria.			6	A verdade	não	poupa	nem	as celebridades.		
	25	26	27	28	29				25	26	27	28	29		
7	Entre	com seu	código	e número	da conta.			7	O baile	começa	depois	da banda	chegar.		
	30	31	32	33	34				30	31	32	33	34		
8	É	de fundamental	importância	encontrar	uma	solução	comum.	8	Apesar	desse	resultado	tomarei	uma	decisão.	
	35	36	37	38	39	40	41		35	36	37	38	39	40	
9	Minha	filha	não	foi	à aula	de violão.		9	O peixe	pulou	algumas	vezes	no rio.		
	42	43	44	45	46	47			41	42	43	44	45		
10	O barraco	pode	desabar	em algumas	horas.			10	A apresentação	foi	cancelada	por causa	da chuva.		
	48	49	50	51	52				46	47	48	49	50		
11	O jantar	foi	farto	e agradou	a todos.			11	O menino	desenhou	o planeta	na parede.			
	53	54	55	56	57				51	52	53	54			
12	A mensalidade	aumentou	mais	que a inflação.				12	A visita	transformou-se	em	uma reunião.			
	58	59	60	61					55	56	57	58			
13	As crianças	brincaram	no natal.					13	Eu	tenho	cinco	presentes	para	você.	
	62	63	64						59	60	61	62	63	64	
14	Novas	metas	surgem	na informática.				14	Nunca	uma	vitória	foi	paga	com tanto	suor.
	65	66	67	68					65	66	67	68	69	70	71
15	A maioria	dos convidados	gosta	de sorvete.				15	A temperatura	deve	ficar	abaixo	de zero.		
	69	70	71	72					72	73	74	75	76		
16	Já	era	tarde,	quando	ele	me abordou.		16	O prato	do dia	é	couve	no tempero.		
	73	74	75	76	77	78			77	78	79	80	81		
17	O termômetro	indica	alta	temperatura.				17	A casa	só	tem	um sofá	confortável.		
	79	80	81	82					82	83	84	85	86		
18	A locomotiva	vem	com mais	carga.				18	É	possível	que ele	esteja	fora	de perigo.	
	83	84	85	86					87	88	89	90	91	92	
19	Meu	tio	fez	essa	viagem	seis	vezes.	19	O almoço	foi	servido	ao ar	livre.		
	87	88	89	90	91	92	93		93	94	95	96	97		
20	Comer	quindim	é	sempre	uma	boa	pedida.	20	Desculpe	se te chamo	de velho.				
	94	95	96	97	98	99	100		98	99	100				
List 7								List 8							
1	Isso	se resolverá	de maneira	tranquila.				1	O acidente	de carro	na rodovia	aumentou	a fila.		
	1	2	3	4					1	2	3	4	5		
2	Fumar	prejudica	a saúde.					2	Hoje	irei	à vila	sem	meu filho.		
	5	6	7						6	7	8	9	10		
3	Daqui	a pouco	a gente	vai	ao baile.			3	O calor	está	agradável	nesse	verão.		
	8	9	10	11	12				11	12	13	14	15		
4	A previsão	é	de muito	nevoeiro	na rodovia.			4	Estou	certo	que mereço	a atenção	dela.		
	13	14	15	16	17				16	17	18	19	20		
5	Ainda	é	uma	boa	temporada	pro	cinema.	5	O panorama	das pessoas	é	desanimador.			
	18	19	20	21	22	23	24		21	22	23	24			
6	O clima	é	ruim	no sul	do estado.			6	A temperatura	é	mais	amena	à noite.		
	25	26	27	28	29				25	26	27	28	29		
7	Ela	e seu	namorado	chato	saem	do carro.		7	O maior	mamífero	fica	debaixo	da água.		
	30	31	32	33	34	35			30	31	32	33	34		
8	O musical	levou	quatro	meses	para	ficar	pronto.	8	O sinal	emitido	é	captado	pelos	receptores.	
	36	37	38	39	40	41	42		35	36	37	38	39	40	
9	A balsa	é	o meio	de transporte	daqui.			9	Ela	teria	sido	a melhor	bailarina	do festival.	
	43	44	45	46	47				41	42	43	44	45	46	
10	Os meninos	ganharam	um filhote	de gato.				10	O ministério	mudou	demais	com a eleição.			
	48	49	50	51					47	48	49	50			
11	A ação	se passa	em uma	cidade	calma.			11	Temos	expectativa	de que tudo	fique	tranquilo.		
	52	53	54	55	56				51	52	53	54	55		
12	O candidato	buscava	apoio	eleitoral.				12	A mudança	é	lenta	e duradoura.			
	57	58	59	60					56	57	58	59			
13	Essa	chuva	não	ocorre	mais	todo	ano.	13	O armário	da casa	da minha	avó	é	antigo.	
	61	62	63	64	65	66	67		60	61	62	63	64	65	
14	A sombra	perto	do rio	é	muito	boa.		14	O estilete	é	uma	arma	perigosa.		
	68	69	70	71	72	73			66	67	68	69	70		
15	O telejornal	começa	às dez	da noite.				15	Ele	tem	a meta	de subir	o morro	de bicicleta.	
	74	75	76	77					71	72	73	74	75	76	
16	Meu	time	se consagrou	como	campeão	estadual.		16	O vão	da plataforma	é	estreito.			
	78	79	80	81	82	83			77	78	79	80			
17	A proposta	foi	inspecionada	pela	gerência.			17	Minha	tia	abriu	a caixa	de correspondência.		
	84	85	86	87	88				81	82	83	84	85		
18	O pássaro	canta	ao amanhecer.					18	O menu	inclui	um prato	muito	saboroso.		
	89	90	91						86	87	88	89	90		
19	A juventude	tinha	que revolucionar	a escola.				19	A cantora	terá	quatro	meses	de ensaio.		
	92	93	94	95					91	92	93	94	95		
20	Seu	limite	do cartão	foi	aumentado.			20	O time	continua	lutando	pelo	sucesso.		
	96	97	98	99	100				96	97	98	99	100		
List 9								List 10							
1	Esses	são	nossos	antigos	vizinhos.			1	Neste	caso,	dormirei	tranquilo.			
	1	2	3	4	5				1	2	3	4			
2	O inspetor	faz	a vistoria	completa.				2	Preciso	pegar	uma	caneta	amarela.		
	6	7	8	9					5	6	7	8	9		
3	A aula	dele	é	bastante	interessante.			3	Ele	dorme	num	leito	de palha.		
	10	11	12	13	14				10	11	12	13	14		
4	O congresso	volta	atrás	em sua	palavra.			4	Procurei	minha	amiga	em casa.			
	15	16	17	18	19				15	16	17	18			
5	O mau	tempo	finalmente	chegou	na serra.			5	O grêmio	ganhou	uma	quadra	de esportes.		
	20	21	22	23	24				19	20	21	22	23		
6	A explicação	pode	ser	encontrada	na tese.			6	A empresa	tem	uma	grande	produção	de metal.	
	25	26	27	28	29				24	25	26	27	28	29	
7	Os móveis	para	o quarto	chegarão	às três	da tarde.		7	Hoje	irei	precisar	de você.			
	30	31	32	33	34	35			30	31	32	33			
8	Durante	o dia	apague	a luz.				8	O dia	está	bom	para	passear	no parque.	
	36	37	38	39					34	35	36	37	38	39	
9	O clima	está	muito	seco	no interior.			9	Sem ele	o tempo	flui	num	ritmo	suave.	
	40	41	42	43	44				40	41	42	43	44	45	
10	Elas	traziam	o equipamento.					10	Ela	organizou	um grande	banquete.			
	45	46	47						46	47	48	49			
11	O trabalho	se tornou	cansativo	para	os alunos.			11	Ainda	não	se sabe	o dia	da prova.		
	48	49	50	51	52				50	51	52	53	54		
12	O pão	que eu	comprei	era	ótimo.			12	Meu	pai	gosta	de dormir	cedo	durante	a semana.
	53	54	55	56	57				55	56	57	58	59	60	61
13	Nossa	filha	foi	a primeira	a se formar	no exterior.		13	A paixão	dele	é	a natureza.			
	58	59	60	61	62	63			62	63	64	65			
14	Hoje,	eu	não	pude	fazer	minha	ginástica.	14	Eu	gosto	de tomar	banho	gelado	pela	manhã.
	64	65	66	67	68	69	70		66	67	68	69	70	71	72
15	A menina	estava	em cima	da escada.				15	Será	muito	difícil	conseguir	que eu	coma.	
	71	72	73	74					73	74	75	76	77	78	
16	O comércio	daqui	é	bem	tranquilo.			16	A intenção	é	ter	o apoio	do governador.		
	75	76	77	78	79				79	80	81	82	83		
17	Eu	tive	uma	prova	fácil	de geografia.		17	Desculpe,	mas	me atrasei	no casamento.			
	80	81	82	83	84	85			84	85	86	87			
18	O frio	deve	diminuir	ainda	este	ano.		18	O caminho	até	a fazenda	é	perigoso		
	86	87	88	89	90	91			88	89	90	91	92		
19	A médica	orientou	que eles	mudassem	o remédio.			19	A escuridão	do quarto	assutou	a criança.			
	92	93	94	95	96				93	94	95	96			
20	Nossas	atitudes	são	calmas.				20	O jogo	será	transmitido	à tarde.			
	97	98	99	100					97	98	99	100			

In the pilot study, carried out with three normal, male listeners with an average age of 23.33 years, the average sentence recognition index was 99.16%.

The first participant in the pilot study correctly answered 99.9% of the test, with only one error in List 1. The second participant correctly answered98.8%, with nine errors in List 3, one error in List 4, one error in List 5, and one error in List 9, totaling 12 errors. The third individual correctly answered99.3% of the test, with six errors in List 5 and one error in List 7, totaling seven errors. Errors made by the participants involved a lack of attention in presenting the sentence, leading to non-repetition of the sentence, or word changes, or even changes of words from plural to singular and, in one case, the reduction of *“para a'* to ‘*pra'* (used in colloquial speech).

## DISCUSSION

Of the 200 sentences analyzed in this study, the first stage reviewers suggested modifying71 sentences (35.5%). The internal reliability coefficient was high for both different and common sentences, showing that there is an agreement between the reviewers for the evaluated criteria. This data is very important for the more reliable selection of the speech material to be produced.

Predictability was the criterion most frequently cited by the reviewers as requiring sentence modification (Figure 1), with the average score for this criterion being 52.58% for different sentences and 56.76% for sentences in common. Comparing the mean of the answers in the criteria evaluated by the reviewer subgroups, we found that there was greater variation in the answers for the predictability criterion (Table 1 and 2).

For the predictability criterion, the closer to zero the score, the less predictable the sentence. In the literature, we observed that this criterion was also used to construct other materials for speech perception assessment in normally hearing adults^(24)^ and elderly people with and without hearing loss^(16)^.

Predictability implies that the keyword, normally located at the end of the sentence, is predictable due to the presence in the sentence of other words semantically linked to it. Low predictability, means that the prediction of the keyword from the context is not possible, due to an absence of other words in the sentence that are semantically linked to it^(16)^. Thus, the less predictable the sentence, the more reliable the speech recognition assessment will be.

A national study^(16)^ with elderly people, with and without hearing loss, used more or less predictable sentences to assess speech recognition with silence and noise. This study revealed that elderly individuals under silent testing conditions performed better than those under noisy conditions. This study also reported that elderly individuals with greater hearing loss, indicated more benefit stemming from context support. Data from the reported study show the importance of considering the predictability item in the construction of speech perception tests.

Another study^(24)^ carried out with normal hearing individuals, which aimed to assess speech recognition, analyzed the influence of predictability of words using sentences with low and high predictability. The study found that the punctuation differences between the two types of sentences indicate the degree to which the listener can benefit from semantic, syntactic, and prosodic information provided by the sentence context, that is, the use of these sentences indicates the extent to which a person can use the context.

The therapist group was the one that recommended the largest number of modifications based on the predictability criterion. The linguist group, on the other hand, based its modification recommendations on familiarity and sentence meaning criteria. We believe that speech-language therapists, possibly because they deal with the experience of hearing-impaired patients in their clinical practice, are more inclined to take the predictability factor into account. With the decline of auditory and cognitive functions, their patients struggle to understand the information to be memorized, thereby becoming more dependent on word intelligibility and the linguistic context of the sentence for support^(25)^.

On the other hand, linguists analyze from a lexical point of view, considering words as isolated elements. Given this, the degree of familiarity with each word and its meaning are the particularities most considered for the selection and control of materials by this group^(26)^. Therefore, we see the importance of approaching these two groups from distinct perspectives, to be able to more effectively update the sentence lists.

Familiarity is the frequency with which a certain linguistic input is heard and which input is used, that is, how much the expression is known^(27)^. We found that this criterion is also applied when constructing speech materials used with adults^(6)^ and normal-hearing children^(4,5)^, adults using CI^11^, elderly people with and without hearing loss^(16)^, and in children, adolescents, and adults who are native speakers of BP^(28)^.

The familiarity criterion is essential in the construction of speech perception tests, especially those intended for children, since the use of words unknown by the child can generate suboptimal outcomes in the auditory recognition of speech sounds.

A study^(5)^ that aimed to apply speech perception material in a closed setting to analyze the percentage rate of speech recognition (PRSR) in children with hearing impairments found that the familiarity and sentence meaning criteria are as important as the chosen speech stimulus.

The number of sentences that needed to be modified following the evaluation of the first stage reviewers (35.5%) to make the speech bank more reliable and applicable in clinical practice was reduced. This result shows that this speech bank, already revised in 1998, has content that, in addition to being phonetically balanced, is familiar, with clear meaning and low predictability.

Reviewer experience was an aspect that helped to update the sentences. We found that both linguists and therapists had, in general, graduate degrees in the area of interest for this research (84.21%). Therapists had an average of 17 years-experience in the area of auditory rehabilitation, including experience in applying speech perception tests. Additionally, linguists typically had more than one area of expertise, most in the areas of phonetics and phonology - which are of great importance for studies regarding the production and perception of speech sounds^(29)^.

Most therapist reviewers in this study use speech recognition tests in their clinical practice (75%). A descriptive study^(13)^ carried out on these speech tests used in cochlear implant centers in Brazil found that 63% of the services evaluated apply the tests in the therapeutic context and that there is no uniformity in the assessment procedures with the use of speech perception tests.

In addition to the 71 sentences recommended for modification by the reviewers at the first stage of the study, reviewers at the second stage also recommended a further 28 sentences for modification. Considering the modifications from the first (71 sentences) and second (28 sentences) stages, a total of 99 sentences (49.50%) from the original list were modified to prepare a list of sentences aimed at evaluating the speech perception of individuals and suitable for the clinical context.

In the second stage, the proper name was the element that was most indicated for modification in the sentences. We believe that the literature recommends the exclusion of proper names in sentences to avoid regionalism, since some names may not be familiar or common, making translation into other languages difficult.

Another criterion adopted in this study was the verification of whether sentences had no more than seven phonological words. Controlling the number of words has been recommended since 1955^(22)^ to prevent memory access interfering with speech recognition assessment. In this study, the author recommends that sentences have no more than 12 words. A1979^(23)^ study recommended that sentences should not exceed seven syllables. A national study^(7)^ that used the criteria of both studies cited considered that sentences should have four to seven phonological words.

Another study reported that in the assessment of working memory in people with hearing loss, the phonological loop is accessed. In individuals with impaired auditory sensory functions, there may be problems accessing the phonological loop and processing auditory information. To compensate for this decreased perception, patients with CI depend more on top-down processing that uses phonological/lexical access and long-term memory storage^(30)^.

Thus, word control is very important in sentences to avoid cognitive strain in these individuals, which could lead to mistakes in speech recognition tests.

In addition to limiting the number of phonological words per sentence, the control of the number of phonological words per list was also carried out. We observed that in another widely used speech recognition test^(7)^, especially for cochlear implant users, the lists are made up of 50 phonological words. In the present study, 100 phonological words per list were chosen so that they can be used both to calculate the percentage of the speech recognition index, and the speech recognition threshold. For this same reason, in this study, we decided to leave a greater number of sentences per list than the original study, which had only 10 sentences per list^(20)^. In the literature, we found that other more recent speech recognition tests, such as the HINT^(8)^, present the same number of sentences per list as this study.

In the pilot study carried out to assess the clinical viability of the lists, we found that the scores of the participants were very close to 100% correct. None of the participants reported difficulties in performing the test. We believe that individuals showed excellent speech recognition because, in addition to the lists presenting content with good meaning and familiarity, care was taken to control the number of phonological words per sentence to avoid attentional and/or memory issues, as recommended in the literature ^7.21, 22,30^.

The next stage of this research will be to record sentences in the studio and use the recorded sentences in trials with normal hearing subjects and individuals with different degrees of hearing loss, seeking to standardize the speech recognition threshold under silent and noisy conditions. For that, the recorded sentences will be inserted into the perSONA software, developed by the Laboratory of Vibrations and Acoustics at UFSC, allowing the assessment of speech perception with competitive noise, and thereby reproducing complex static and dynamic acoustic contexts.

This study was of great importance as it shows the relevance of updating speech recognition tests for everyday life and contributing to the creation of reliable material for clinical practice that can help standardize the assessment of speech perception in Brazil with normal listeners and with individuals with different degrees of hearing loss.

## CONCLUSION

After analyzing the data and results obtained, we consider that this study achieved its objective, that is, to adapt a list of sentences to assess speech recognition for adults who speak BP.

It was possible to update the sentences in the speech bank, with the predictability criterion being the most indicated by the reviewers in the first stage of the study. In the second stage, the exclusion of proper names and sentences that contained excessive abstraction or inadequate semantics, was the most recommended.
