# Supplementary material for: Updating sentences lists for assessment speech perception
Source: Codas. 2022 Jan 10;34(1):e20200301. doi: 10.1590/2317-1782/20202020301 (PMC9886115; doi:10.1590/2317-1782/20202020301)
Supplement: Translation

# Translation: Adaptação de listas de sentenças para avaliação da percepção da fala

**Authors:** Maria Madalena Canina Pinheiro, Michele Gindri Vieira, Lara Malafaia Vieira, Isadora Koerich, Isadora Rosseto, Cristiane Lazzarotto-Volcão, Stephan Paul

**Affiliations:** 1 Departamento de Fonoaudiologia, Universidade Federal de Santa Catarina – UFSC - Florianópolis (SC), Brasil.; 2 Centro Catarinense de Reabilitação/Centro Especializado em Reabilitação, Secretaria de Estado da Saúde de Santa Catarina- Florianópolis (SC), Brasil.; 3 Curso de Fonoaudiologia, Universidade Federal de Santa Catarina – UFSC - Florianópolis (SC), Brasil.; 4 Departamento de Língua e Literatura Vernáculas, Universidade Federal de Santa Catarina – UFSC - Florianópolis (SC), Brasil.; 5 Departamento de Engenharia Mecânica, Universidade Federal de Santa Catarina – UFSC - Florianópolis (SC), Brasil.

**Keywords:** Percepção da Fala, Percepção Auditiva, Audição, Testes de Discriminação da Fala, Testes Auditivos

## Abstract

**Objetivo:**

Adaptar listas de sentenças para avaliar o reconhecimento de fala em adultos.

**Método:**

Foram atualizadas 200 sentenças balanceadas foneticamente que passaram por duas etapas de revisão. Na primeira etapa, foi enviado um questionário *on-line* para 60 juízes analisarem as sentenças em relação aos critérios de familiaridade, significado e previsibilidade. Para análise da consistência interna do questionário foi aplicado coeficiente Alfa de Cronbach. Na segunda etapa, três juízes especialistas analisaram se as mesmas estavam de acordo com os parâmetros indicados pela literatura para a construção de sentenças e organizaram em 10 listas de 20 sentenças cada, a fim de facilitar a avaliação clínica do reconhecimento de fala. Foi realizado um estudo piloto com três indivíduos jovens e normo-ouvintes.

**Resultados:**

Na primeira etapa foram analisadas as respostas de 15 juízes que preencheram todo o questionário. Verificou-se que a concordância entre os juízes foi alta para todos os critérios. Foram indicadas 71 sentenças para serem modificadas na primeira etapa, sendo a previsibilidade o critério que teve maior ocorrência de modificação. Na segunda etapa foram identificadas mais 28 sentenças passíveis de ajustes, sendo a presença de nome próprio o critério mais frequente. No estudo piloto os jovens apresentaram alto índice de reconhecimento de fala.

**Conclusão:**

Concluiu-se que a maioria das modificações realizadas nas sentenças deste estudo possibilitou a criação de um material fidedigno para a prática clínica fonoaudiológica que contribuirá na padronização da avaliação da percepção da fala de indivíduos normo-ouvintes e com perda auditiva.

## INTRODUÇÃO

A habilidade de compreensão é fundamental para a integração social, pois é o primeiro passo que possibilita a comunicação entre os indivíduos. Essa habilidade é considerada o aspecto mais importante a ser medido na função auditiva gerando dados que mostram como o indivíduo escuta e entende em sua rotina diária^(1)^.

A aproximação da Fonoaudiologia com a Linguística desenvolveu técnicas para diagnosticar precocemente alterações da linguagem dos indivíduos, bem como para desenvolver protocolos de avaliação da compreensão da fala. O campo de processamento da fala na Engenharia vem evoluindo constantemente, contribuindo com recursos tecnológicos que possibilitaram aprimorar, padronizar e automatizar testes utilizados na área audiológica^(2)^. Por meio dessa relação ocorreu um grande avanço na avaliação da percepção da fala^(3)^.

Na literatura, verificaram-se materiais de fala que já foram desenvolvidos com o objetivo de avaliar a detecção e a discriminação de sons e palavras^(4,5)^, além da habilidade de reconhecimento de monossílabos, palavras e sentenças^(6-8)^.

Para a avaliação da comunicação real, a literatura recomenda a aplicação de sentenças com ruído competitivo^(8-11)^. Essas sentenças podem ser utilizadas tanto em normo-ouvintes^(12)^, quanto em indivíduos com perda auditiva^(6)^. No entanto, sua maior aplicação ocorre para avaliação e acompanhamento de candidatos e usuários de aparelhos de amplificação sonora individual (AASI) e de implante coclear (IC)^(13,14)^.

Com a análise da literatura nacional verificou-se que na prática clínica atualmente, há poucas opções de testes de percepção de fala disponíveis para utilizar na avaliação de adulto.,Nesse contexto, um teste muito conhecido é o Lista de Sentenças em Português^(6)^, que está disponível em *Compact Disc.*Esse teste necessita que o próprio fonoaudiólogo ajuste os níveis de fala e ruído. Há várias pesquisas que utilizam esse material e reconhecem sua grande aplicabilidade^(13,15,16)^.

Outro teste para avaliar o reconhecimento da fala é o Listas de Sentenças do Centro de Pesquisas Audiológicas (CPA), que utiliza sentenças balanceadas foneticamente e é muito utilizado na maior parte dos serviços de IC. Porém, o mesmo é utilizado na maior parte dos serviços à viva-voz, não havendo uma uniformidade nas condutas de aplicação do mesmo^(7)^.

Um teste que vem sendo muito utilizado, especialmente na área de pesquisas, é o *Hearing in Noise Test* (HINT)^(8)^. Ele tem a vantagem de ser reconhecido internacionalmente e a desvantagem do alto custo da sua licença para uso na prática clínica^(17)^.

Com o intuito de disponibilizar para a prática fonoaudiológica um novo teste de reconhecimento de fala, foram analisados bancos de fala doportuguês brasileiro (PB). Os bancos de fala são arquivos compostos por um grande número de sentenças que possibilitam captar as variações e mudanças em uma comunidade de fala^(18,19)^.

Um banco de fala muito conhecido no Brasil foi desenvolvido por Alcaim^(12)^ e adaptado por Seara^(20)^. Esse banco é constituído por sentenças balanceadas foneticamente, as quais contêm 35 segmentos (fonemas e suas variações) do PB com ocorrência total de 20.178 sons ao longo das sentenças.

A pesquisa realizada por Seara apresentava finalidades acadêmicas e destacou-se por um rigoroso cuidado com o balanceamento fonético, o qual foi mais completo que o autor proponente das mesmas^(12)^. No entanto, devido às finalidades clínicas do presente estudo, bem como a preocupação em adequar o vocabulário, o significado e a previsibilidade do material de fala, verificou-se a necessidade de atualização das sentenças.

Com o intuito de disponibilizar para a prática clínica um teste que avalie o reconhecimento de fala e facilite a atuação do fonoaudiólogo, o presente estudo teve o objetivo geral de adaptar uma lista de sentenças para avaliar o reconhecimento de fala em adultos.

## MÉTODO

A pesquisa foi de caráter observacional, transversal e analítico, sendo que ocorreu no período de junho a agosto de 2019. A mesma foi aprovada pelo Comitê de Ética em Pesquisa da Universidade Federal de Santa Catarina (UFSC) e tem como número de aprovação do Parecer: 1.997.931 e CAAE56838816.7.0000.0121.

No presente trabalho foi proposto adaptar listas de sentenças para avaliar o reconhecimento de fala, com base em um banco de fala com sentenças balanceadas foneticamente, adaptado por Seara^(20)^ e de autoria de Alcaim^(12)^. O banco de fala de Seara^(20)^ é composto por 200 sentenças distribuídas igualmente em 20 listas com 10 sentenças cada. Essas sentenças são muito utilizadas como banco de fala em ensaios de inteligibilidade de fala com normo-ouvintes na área da Engenharia.

O presente estudo foi realizado em três etapas: análise dos juízes por meio de um questionário on-line, atualização das sentenças de acordo com os critérios de construção fundamentados pela literatura e estudo piloto com normo-ouvintes.

### 1ª etapa - Análise dos juízes por meio de questionário on-line

Nesta etapa foi construído um questionário *on-line* para os juízes analisarem as sentenças de Seara^(20)^.

As sentenças do banco de fala foram transportadas para uma plataforma *on-line* (*SurveyMonkey*), visando a facilitar o acesso e a participação de juízes de várias regiões do país.

Para realizar a análise das sentenças, foram convidados dois grupos de juízes constituídos por linguistas e fonoaudiólogos com experiência em reabilitação auditiva. Os linguistas convidados deveriam ter no mínimo mestrado em sua formação acadêmica e os fonoaudiólogos reabilitadores deveriam ter no mínimo três anos de experiência. Esses profissionais foram indicados por professores e profissionais com experiência nas áreas de fonética, fonologia e reabilitação auditiva. Antes de enviar o questionário *on-line* os juízes foram contatados por *e-mail* ou rede sociais.

O grupo dos linguistas foi denominado GL e foi subdividido em dois grupos (GL1 e GL2). O grupo dos reabilitadores auditivos foi denominado GR, sendo subdividido em GR1 e GR2.

Devido ao grande número de sentenças, optou-se por dividir 187 sentenças em quatro subgrupos para facilitar a análise, sendo enviadas 47 sentenças para os subgrupos GL1, GL2 e GR2 e 46 para o subgrupo GR1. Além destas sentenças diferentes, cada subgrupo analisou também, 13 sentenças em comum escolhidas aleatoriamente a fim de analisar a concordância de todos os subgrupos.

Anteriormente à análise das sentenças, os juízes deveriam responder questões sobre sua formação em nível de graduação e pós-graduação, seu vínculo institucional, se possuíam experiência em análise acústica e qual sua área de estudo. Aos reabilitadores foram feitos questionamentos sobre o ano de conclusão da graduação em Fonoaudiologia, o tempo de trabalho como reabilitador auditivo e se aplicavam testes de reconhecimento de fala.

Na análise das sentenças cada juiz deveria dar uma classificação de notas para os seguintes critérios: familiaridade, significado de sentença e previsibilidade. As respostas foram coletadas por meio de escalas de linha contínua, implementadas no SurveyMonkey por meio de uma barra de deslizar que variava de zero (0) a cem (100).

A classificação da familiaridade de cada sentença foi feita de acordo com o quão comuns ou conhecidas eram as sentenças para o juiz. Neste item, a nota 0representava que a sentença não era comum ou conhecida para o juiz e a nota 100, muito comum ou conhecida.

O significado da sentença foi julgado de acordo com o sentido mobilizado pela sentença. Neste item, a nota zero representa ausência de sentido e a nota 100, sentido completo.

A previsibilidade foi classificada de acordo com a expectativa de continuação da sentença a partir de algumas palavras iniciais. A nota 0representa nenhuma expectativa do que poderia complementar a sentença e a nota 100, a possibilidade de prever o final lendo somente o início da sentença, ou seja, apresentava muita previsibilidade.

Ao final da primeira etapa, os dados foram analisados de forma descritiva e, para verificar a consistência interna das respostas do questionário aplicado aos juízes, foi utilizado o teste Cronbach's Alpha calculado pelo software SPSS.O coeficiente Alfa de Cronbach's mensura a relação entre as respostas de um questionário, por meio da análise das respostas dos participantes da pesquisa. Esse coeficiente reflete o grau de covariância entre os itens de uma escala, apontando que quanto menor a soma da variância dos itens, mais consistente é considerado o instrumento. Valores maiores ou iguais a 0,70 são indicativos de consistência interna adequada^(21)^.

### 2ª etapa - Atualização das sentenças de acordo com os critérios de construção fundamentados pela literatura

Na segunda etapa da pesquisa, três juízes que não participaram da primeira análise atualizaram as sentenças apontadas pelos juízes da primeira etapa, executando as modificações indicadas quanto aos critérios de familiaridade, significado de sentença e previsibilidade. Os juízes da segunda etapa eram dois doutores na área de linguística e um doutor na área de reabilitação auditiva.

Somado a isso, os juízes da segunda etapa verificaram se as mesmas estavam de acordo com parâmetros de construção de sentenças fundamentados pela literatura^(6,22,23)^, a saber: exclusão dos nomes próprios; sentenças afirmativas com período simples e composto; sentenças constituídas de três a sete vocábulos fonológicos e com baixo nível de abstração. Ainda, nesta segunda etapa, as modificações em cada sentença também priorizaram o aspecto semântico em detrimento ao fonético, para adequação das sentenças ao objetivo do trabalho.

Considerou-se vocábulo fonológico a palavra fonológica ou prosódica que não tem mais do que um acento primário (tônico). Este critério foi utilizado para viabilizar a contagem do número de acertos na aplicação do teste de reconhecimento de fala.

Após serem realizadas estas adequações, as sentenças foram organizadas em 10 listas com 20 sentenças cada, também a fim de facilitar a avaliação clínica do reconhecimento de fala. Ainda foi analisado, em cada uma das listas de 20 sentenças, o número de sentenças modificadas pelos juízes e as consideradas originais (em relação ao trabalho de Seara), a fim de que houvesse uma distribuição mais uniforme em cada lista, mantendo o máximo possível do balanceamento fonético por lista.

A seguir, observou-se a necessidade de adequação no número de vocábulos fonológicos por lista, visando à obtenção de 100 vocábulos fonológicos em cada uma das 10 listas. Para isso, as listas foram revisadas e as adequações foram realizadas nas sentenças que já haviam sido indicadas para modificações pelos juízes da primeira etapa. Dessa forma, as sentenças originais não foram alteradas.

No final da revisão, cada lista apresentou 100 vocábulos fonológicos, sendo cada vocábulo fonológico da lista correspondente a 1%, ou seja, o número de erros de vocábulos fonológicos obtidos pelo paciente em uma determinada lista será diminuído da pontuação total (100%) e o resultado será expresso em porcentagem.

### 3ª etapa - Estudo piloto com normo-ouvintes

Para avaliar a aplicabilidade clínica das listas de sentenças, foi realizado um estudo pilotocom três indivíduos jovens (média de idade de 23,33 anos), normo-ouvintes, sem queixas atencionais e/ou de memória,no Laboratório de Vibrações e Acústicas do Departamento de Engenharia Mecânica da UFSC. Durante a aplicação das listas, o indivíduo permaneceu com fone circum-auralSennheiserHDA200,em uma cabina acústica e foi utilizado um audiômetro modelo AC 40 da marca da Interacoustics. As 200 sentenças foram apresentadas a viva voz, em uma intensidade fixa de 50 dBNA bilateralmente, controladas pelo VU meter. O mesmo avaliador aplicou todas as listas nos indivíduos num mesmo dia, sendo realizados intervalos para evitar fadiga.

A análise dos dados da segunda e terceira etapa foi descritiva.

## RESULTADOS

As sentenças deste estudo foram enviadas na primeira etapa por meio de um questionário *on-line* para 60 juízes, sendo 37 linguistas e 23 reabilitadores. No grupo de linguistas (GL), 26 juízes abriram o questionário, mas apenas sete (18,91%) analisaram todos os itens do questionário. No grupo de reabilitadores (GR), 21 juízes abriram o questionário, sendo que oito (34,78%) analisaram todos os itens do questionário. A amostra final resultou nas avaliações completas de 15 juízes, sendo sete linguistas e oito reabilitadores.

Com relação à formação inicial dos sete indivíduos do GL, pôde-se observar que a maioria era fonoaudiólogo (42,85%), seguido pelo curso de Letras-Português-Espanhol (28,59%), Letras-Inglês (14,28%) e Engenharia Elétrica (14,28%). Destes participantes, a maioria tinha formação em pós-graduação em Linguística (42,85%).

No GR os oitos indivíduos (100%) eram fonoaudiólogos com média de 19 anos e meio de formação e com média de 17 anos e meio de experiência na área de reabilitação auditiva. A respeito da experiência em aplicação de testes de percepção de fala, seis (75%) responderam que aplicavam testes de reconhecimento de fala.

O GL informou, no questionário, sobre a experiência em análise acústica prévia, sendo que seis (85,70%) juízes apresentaram experiência em análise acústica e um (14,30%) não preencheu a pergunta.

A Tabela 1 apresenta a estatística descritiva das classificações das 13 sentenças em comum analisadas pelos grupos GL e GR.

**Tabela 1:** Análise descritiva das respostas dos juízes para os critérios familiaridade, significado de sentença e previsibilidade nas sentenças em comum

		Média	Mediana	Desvio Padrão	Máximo	Mínimo
Familiaridade	GL	86,10%	90,37	10,94	100,00	0,00
	GR	84,38%	83,75	10,12	100,00	0,00
	Total	85,24%	87,50	10,47	100,00	0,00
Significado de Sentença	GL	90,39%	92,50	6,68	100,00	35,00
	GR	90,01%	89,83	7,11	100,00	25,00
	Total	90,20%	90,75	6,83	100,00	25,00
Previsibilidade	GL	48,21%	45,75	16,13	100,00	0,00
	GR	65,31%	64,54	18,76	100,00	0,00
	Total	56,76%	54,87	19,35	100,00	0,00

Legenda: GL = Grupo de Linguistas; GR = Grupo de Reabilitadores

O critério previsibilidade é considerado o inverso da familiaridade e do significado de sentença, pois para este critério a nota zero representava baixa previsibilidade enquanto nos itens familiaridade e significado de sentença a nota máxima 100 representava muita familiaridade ou sentença com sentido totalmente completo.

Observou-se na Tabela 1 que os dois grupos produziram respostas similares em relação aos critérios familiaridade e significado da sentença e que o grupo de reabilitadores julgou as sentenças mais previsíveis do que os linguistas. O coeficiente Cronbach's Alpha revelou que para análise das 13 sentenças em comum a concordância dos juízes em relação aos parâmetros previsibilidade (0,986), familiaridade e significado de sentença (0,960) foi alta.

Em relação à análise das 187 sentenças, julgadas pelos subgrupos, o coeficiente Cronbach's Alpha mostrou que a concordância encontrada em relação aos critérios de previsibilidade para o grupo de linguistas foi de 0,99 e para grupo de reabilitadores de 0,97. Para o parâmetro de familiaridade e significado de sentença foi de 0,93 para o grupo de linguistas e de 0,92 para grupo de reabilitadores.

Foi realizada a análise dos subgrupos separadamente a respeito das respostas dos juízes no questionário *on-line*. Na Tabela 2, pode-se verificar a análise descritiva dos juízes com relação às 47 sentenças avaliadas por cada subgrupo frente a cada critério.

**Tabela 2:** Análise descritiva das respostas dos juízes por subgrupos para os critérios familiaridade,significado de sentença e previsibilidade das sentenças diferentes

		Média	Mediana	Desvio Padrão	Máximo	Mínimo
Familiaridade	GL1	90,10%	95,00	11,56	100,00	10,00
	GL2	80,43%	80,25	12,47	100,00	7,00
	GR1	80,57%	83,33	15,92	100,00	0,00
	GR2	82,15%	83,33	10,96	100,00	18,00
	Total	83,33%	86,66	13,36	100,00	0,00
Significado de Sentença	GL1	87,53%	93,50	13,10	100,00	20,00
	GL2	87,62%	89,50	9,97	100,00	11,00
	GR1	85,49%	87,12	13,33	100,00	0,00
	GR2	86,25%	89,00	12,12	100,00	23,00
	Total	86,73%	89,50	12,13	100,00	0,00
Previsibilidade	GL1	29,65%	31,25	13,90	100,00	0,00
	GL2	55,95%	55,50	12,67	100,00	0,00
	GR1	47,84%	47,87	12,43	100,00	0,00
	GR2	76,77%	78,00	13,28	100,00	17,00
	Total	52,58%	53,50	21,39	100,00	0,00

Legenda: GL1 = Grupo de Linguistas 1; GL2 = Grupo de Linguistas 2; GR1 = Grupo de Reabilitadores 1; GR2 = Grupo de Reabilitadores 2

Verificou-se na Tabela 2 que o subgrupo GL1 foi o que atribuiu notas mais altas as sentenças quanto à familiaridade. Nos demais subgrupos a média de julgamento foi similar. Também pode-se perceber que o GL1 foi o que considerou as sentenças com menor previsibilidade e o GR2 considerou as sentenças mais previsíveis. Verificou-se que no julgamento da previsibilidade houve maior desvio padrão nas respostas dos subgrupos se comparado aos critérios familiaridade e significado da sentença.

A partir destas análises, das 187 sentenças diferentes, optou-se por modificar as que tiveram o julgamento dos critérios familiaridade abaixo de 69,97% (Média 83,33 - DP 13,36), significado das sentenças abaixo de 74,60 (Média 86,73 - DP 12,13) e/ou acima de 73,97% (Média 52,58% + DP 21,39) para a previsibilidade.

O limiar percentual que verificou necessidade de modificação das 13 sentenças analisadas por todos os subgrupos, foi calculado separadamente. Optou-se por modificar as que tiveram o julgamento dos critérios familiaridade abaixo de 74,77% (Média 85,24 - DP 10,47), significado das sentenças abaixo de 83,37 (Média 90,20 - DP 06,83) e/ou acima de 76,12% (Média 56,76% + DP 19,35) para a previsibilidade.

As Figuras 1 e 2 ilustram a quantidade de sentenças julgadas pelos critérios pré-estabelecidos de previsibilidade, familiaridade, significado de sentença e em critérios concomitantes, que precisarão ser modificadas com base nos critérios e acima descritos, sendo a 1 referente às sentenças diferentes e a 2 às sentenças em comum. Na Figura 1 observa-se a análise da quantidade de sentenças julgadas por cada subgrupo de juízes.

**Figura 1:** Distribuição da quantidade das sentenças diferentes a serem modificadas por cada subgrupo de juízes segundo os critérios avaliados

Legenda: GL1 = Grupo de Linguistas 1; GL2 = Grupo de Linguistas 2; GR1 = Grupo de Reabilitadores 1; GR2 = Grupo de Reabilitadores

**Figura 2:** Distribuição da quantidade das sentenças em comum a serem modificadas segundo os critérios avaliados

Como pode-se observar na Figura 1, o GR2 foi o subgrupo que mais indicou sentenças para modificação no critério previsibilidade e para os critérios familiaridade e significado de sentenças foi o GR1.

Das 187 sentenças diferentes analisadas, 60 (32.00%) sentenças foram modificadas, sendo destas, oito (13,33%) sentenças pelo critério de familiaridade,três (5,00%) sentenças pelo critério de significado de sentença, 30 (50.00%) sentenças pelo critério de previsibilidade, 16 (26,66%) sentenças pelos critérios de familiaridade e significado de sentença em concomitante, uma (1,66%) sentença pelos critérios de significado de sentença e previsibilidade e duas (3,33%) sentenças pelos três critérios.

Já das 13 sentenças que foram analisadas por todos os subgrupos, 11 (84,6%) foram julgadas frente aos critérios indicando necessidade de modificação, sendo uma (9,09%) sentença pelo critério de significado de sentença, três (27,27%) sentenças pelo critério de previsibilidade, quatro (36,36%) sentenças pelos critérios de familiaridade e significado de sentença em concomitante, uma (9,09%) sentença pelos critérios de familiaridade e previsibilidade em concomitante e duas (18,18%) sentenças pelos critérios de significado de sentença e previsibilidade em concomitante.

Na segunda etapa os juízes identificaram mais 28 sentenças passíveis de modificação segundo parâmetros apontados pela literatura, sendo destas, três sentenças não afirmativas, cinco sentenças que não continham baixo nível de abstração e boa semântica, sete que continham mais de oito vocábulos fonológicos e 13 que continham nomes próprios.

Ainda nesta etapa, foi analisada a quantidade de vocábulos fonológicos por lista. Apenas a lista 7 apresentava 100 vocábulos fonológicos. As listas 1 e 5 excederam a quantidade de vocábulos fonológicos definidos por lista. Já as listas 2, 3, 4, 6, 8 a 10 variaram de 89 a 98 vocábulos por lista. As adequações do número de vocábulos fonológicos por lista para 100, foram realizadas nas sentenças que foram apontadas pelos juízes da primeira etapa para serem modificadas.

A lista completa com as 200 sentenças finais pode ser visualizada no Quadro 1.

**Quadro 1:** Listas do Teste de Reconhecimento de Sentenças

Lista 1								Lista 2							
1	Recebi	meu	pai	para	almoçar.			1	Esse	tema	foi	falado	na conferência.		
	1	2	3	4	5				1	2	3	4	5		
2	Minha	sobrinha	tem	um lindo	casaco.			2	Queremos	discutir	o orçamento.				
	6	7	8	9	10				6	7	8				
3	Os pesquisadores	não	acreditam	nessa	história.			3	Meu	neto	corre	bem	rápido	na rua.	
	11	12	13	14	15				9	10	11	12	13	14	
4	O trabalho	pode	mudar	a vida.				4	Ela	seguia	discretamente.				
	16	17	18	19					15	16	17				
5	Foi	muito	difícil	entender	a canção	de natal.		5	O sorvete	do menino	tem	cobertura	de caramelo.		
	20	21	22	23	24	25			18	19	20	21	22		
6	Nós	nascemos	aqui	neste	hospital.			6	A pesca	é	proibida	nesse	canto.		
	26	27	28	29	30				23	24	25	26	27		
7	A sujeira	lançada	no rio	contamina	os peixes.			7	Aqui	é	um ótimo	lugar	para	tomar	chá.
	31	32	33	34	35				28	29	30	31	32	33	34
8	Meu	voo	foi	marcado	para	às cinco	horas.	8	É hora	do homem	se humanizar	mais.			
	36	37	38	39	40	41	42		35	36	37	38			
9	O candidato	falou	como	se já	estivesse	eleito.		9	O subúrbio	da cidade	tem	muita	história.		
	43	44	45	46	47	48			39	40	41	42	43		
10	Seu	amigo	chegou	na frente	do mercado.			10	A feira	livre	não	funcionará	amanhã.		
	49	50	51	52	53				44	45	46	47	48		
11	O prêmio	será	entregue	na sessão	solene.			11	Cada	sociedade	tem	uma	cultura	única.	
	54	55	56	57	58				49	50	51	52	53	54	
12	A flor	mais	fascinante	fica	aqui.			12	Nosso	filho	ama	os animais.			
	59	60	61	62	63				55	56	57	58			
13	Em muitas	cidades	a população	está	diminuindo.			13	A loja	de produtos	naturais	fica	na próxima	rua.	
	64	65	66	67	68				59	60	61	62	63	64	
14	A inauguração	da praça	é	quarta-	feira.			14	Essa	medida	foi	devidamente	alterada.		
	69	70	71	72	73				65	66	67	68	69		
15	Não	é	permitido	fumar	no interior	do ônibus.		15	A torcida	marcou	a temporada	de jogos.			
	74	75	76	77	78	79			70	71	72	73			
16	O tigre	entrou	no combate.					16	Receba	minha	prima	na festa.			
	80	81	82						74	75	76	77			
17	A cantora	foi	apresentar	um grande	sucesso.			17	Quero	te ver	bem	feliz	quando	ele	voltar.
	83	84	85	86	87				78	79	80	81	82	83	84
18	Eu	viajarei	para	bem	longe	amanhã.		18	A principal	personagem	no filme	é	uma	gueixa.	
	88	89	90	91	92	93			85	86	87	88	89	90	
19	A bolsa	de valores	está	em alta.				19	Seu	crime	foi	ignorado	pela	vítima.	
	94	95	96	97					91	92	93	94	95	96	
20	Perguntei	o horário	com gentileza.					20	A casa	é	enfeitada	com rosas.			
	98	99	100						97	98	99	100			
Lista 3								Lista 4							
1	A casa	foi	vendida	no leilão.				1	Ela	não	tem	fome	quando	sai	de casa.
	1	2	3	4					1	2	3	4	5	6	7
2	Nosso	telefone	está	mudo.				2	A união	dos alunos	rendeu	mudanças.			
	5	6	7	8					8	9	10	11			
3	Dei	dinheiro	para	o menino	comprar	um jogo.		3	Depois	do almoço	te encontro	pro	chá.		
	9	10	11	12	13	14			12	13	14	15	16		
4	Ainda	faltam	seis	minutos.				4	A secretária	não	quer	me dizer	a data.		
	15	16	17	18					17	18	19	20	21		
5	O enfeite	da porta	era	feito	de palha.			5	Faz	um desvio	em direção	ao mar.			
	19	20	21	22	23				22	23	24	25			
6	É	a minha	chance	de esclarecer	a notícia.			6	A pequena	nave	pousou	num	campo	de guerra.	
	24	25	26	27	28				26	27	28	29	30	31	
7	A senhora	sempre	colhe	frutas	maduras.			7	O adiantamento	surpreendeu	a mim	e a todos.			
	29	30	31	32	33				32	33	34	35			
8	Usar	mais	aditivo	pode	ser	desastroso.		8	É	bom	ver	o menino	colhendo	flores.	
	34	35	36	37	38	39			36	37	38	39	40	41	
9	É	fundamental	encontrar	a razão	da existência	humana.		9	Ela	ficou	na fazenda	por uma	hora.		
	40	41	42	43	44	45			42	43	44	45	46		
10	A ideia	é	falha,	mas	interessante.			10	Meu	pai	viajará	de avião	amanhã.		
	46	47	48	49	50				47	48	49	50	51		
11	Um casal	de amigos	saiu	para	festejar.			11	O jardim	exige	muito	trabalho.			
	51	52	53	54	55				52	53	54	55			
12	A saída	para	a crise	dele	é	o diálogo.		12	Eu	venho	jantar	em casa	na quinta-	feira.	
	56	57	58	59	60	61			56	57	58	59	60	61	
13	Esse	filme	parece	bastante	divertido.			13	Hoje	eu	acordei	calmo.			
	62	63	64	65	66				62	63	64	65			
14	Será	bom	que ele	estude	o assunto.			14	Foi	um prazer	conhecer	sua	amiga.		
	67	68	69	70	71				66	67	68	69	70		
15	A garota	foi	no mercado	à noite.				15	A correção	do exame	foi	coerente.			
	72	73	74	75					71	72	73	74			
16	Defender	a ecologia	é	manter	a vida.			16	Fiz	a reserva	para	nosso	passeio	ecológico.	
	76	77	78	79	80				75	76	77	78	79	80	
17	Ele	não	entende	quando	falam	rapidamente.		17	Minha	filha	é	especialista	em música	sacra.	
	81	82	83	84	85	86			81	82	83	84	85	86	
18	A duração	do simpósio	é	de cinco	dias.			18	Os quadros	da parede	mostram	a natureza.			
	87	88	89	90	91				87	88	89	90			
19	O discurso	de abertura	foi	muito	vaiado.			19	Eu	precisei	de tempo	na conferência.			
	92	93	94	95	96				91	92	93	94			
20	A lojinha	não	fica	na esquina.				20	Os hotéis	do litoral	são	fantásticos	para	descansar.	
	97	98	99	100					95	96	97	98	99	100	
Lista 5								Lista 6							
1	O analfabetismo	é	um problema	grave.				1	Ele	caminhou	na praia	calma.			
	1	2	3	4					1	2	3	4			
2	Sei	que amanhã	atingiremos	a meta	proposta.			2	Tenho	muito	orgulho	da nossa	gente.		
	5	6	7	8	9				5	6	7	8	9		
3	Eu	vi	uma	blusa	na loja.			3	Esse	projeto	terá	grande	sucesso.		
	10	11	12	13	14				10	11	12	13	14		
4	Uma	índia	andava	na floresta.				4	Reflita	antes	e discuta	depois.			
	15	16	17	18					15	16	17	18			
5	Meu	irmão	tem	motivos	para	comemorar.		5	Eu	tirei	o título	de eleitor	neste	mês.	
	19	20	21	22	23	24			19	20	21	22	23	24	
6	A corrida	de inverno	foi	uma	alegria.			6	A verdade	não	poupa	nem	as celebridades.		
	25	26	27	28	29				25	26	27	28	29		
7	Entre	com seu	código	e número	da conta.			7	O baile	começa	depois	da banda	chegar.		
	30	31	32	33	34				30	31	32	33	34		
8	É	de fundamental	importância	encontrar	uma	solução	comum.	8	Apesar	desse	resultado	tomarei	uma	decisão.	
	35	36	37	38	39	40	41		35	36	37	38	39	40	
9	Minha	filha	não	foi	à aula	de violão.		9	O peixe	pulou	algumas	vezes	no rio.		
	42	43	44	45	46	47			41	42	43	44	45		
10	O barraco	pode	desabar	em algumas	horas.			10	A apresentação	foi	cancelada	por causa	da chuva.		
	48	49	50	51	52				46	47	48	49	50		
11	O jantar	foi	farto	e agradou	a todos.			11	O menino	desenhou	o planeta	na parede.			
	53	54	55	56	57				51	52	53	54			
12	A mensalidade	aumentou	mais	que a inflação.				12	A visita	transformou-se	em	uma reunião.			
	58	59	60	61					55	56	57	58			
13	As crianças	brincaram	no natal.					13	Eu	tenho	cinco	presentes	para	você.	
	62	63	64						59	60	61	62	63	64	
14	Novas	metas	surgem	na informática.				14	Nunca	uma	vitória	foi	paga	com tanto	suor.
	65	66	67	68					65	66	67	68	69	70	71
15	A maioria	dos convidados	gosta	de sorvete.				15	A temperatura	deve	ficar	abaixo	de zero.		
	69	70	71	72					72	73	74	75	76		
16	Já	era	tarde,	quando	ele	me abordou.		16	O prato	do dia	é	couve	no tempero.		
	73	74	75	76	77	78			77	78	79	80	81		
17	O termômetro	indica	alta	temperatura.				17	A casa	só	tem	um sofá	confortável.		
	79	80	81	82					82	83	84	85	86		
18	A locomotiva	vem	com mais	carga.				18	É	possível	que ele	esteja	fora	de perigo.	
	83	84	85	86					87	88	89	90	91	92	
19	Meu	tio	fez	essa	viagem	seis	vezes.	19	O almoço	foi	servido	ao ar	livre.		
	87	88	89	90	91	92	93		93	94	95	96	97		
20	Comer	quindim	é	sempre	uma	boa	pedida.	20	Desculpe	se te chamo	de velho.				
	94	95	96	97	98	99	100		98	99	100				
Lista 7								Lista 8							
1	Isso	se resolverá	de maneira	tranquila.				1	O acidente	de carro	na rodovia	aumentou	a fila.		
	1	2	3	4					1	2	3	4	5		
2	Fumar	prejudica	a saúde.					2	Hoje	irei	à vila	sem	meu filho.		
	5	6	7						6	7	8	9	10		
3	Daqui	a pouco	a gente	vai	ao baile.			3	O calor	está	agradável	nesse	verão.		
	8	9	10	11	12				11	12	13	14	15		
4	A previsão	é	de muito	nevoeiro	na rodovia.			4	Estou	certo	que mereço	a atenção	dela.		
	13	14	15	16	17				16	17	18	19	20		
5	Ainda	é	uma	boa	temporada	pro	cinema.	5	O panorama	das pessoas	é	desanimador.			
	18	19	20	21	22	23	24		21	22	23	24			
6	O clima	é	ruim	no sul	do estado.			6	A temperatura	é	mais	amena	à noite.		
	25	26	27	28	29				25	26	27	28	29		
7	Ela	e seu	namorado	chato	saem	do carro.		7	O maior	mamífero	fica	debaixo	da água.		
	30	31	32	33	34	35			30	31	32	33	34		
8	O musical	levou	quatro	meses	para	ficar	pronto.	8	O sinal	emitido	é	captado	pelos	receptores.	
	36	37	38	39	40	41	42		35	36	37	38	39	40	
9	A balsa	é	o meio	de transporte	daqui.			9	Ela	teria	sido	a melhor	bailarina	do festival.	
	43	44	45	46	47				41	42	43	44	45	46	
10	Os meninos	ganharam	um filhote	de gato.				10	O ministério	mudou	demais	com a eleição.			
	48	49	50	51					47	48	49	50			
11	A ação	se passa	em uma	cidade	calma.			11	Temos	expectativa	de que tudo	fique	tranquilo.		
	52	53	54	55	56				51	52	53	54	55		
12	O candidato	buscava	apoio	eleitoral.				12	A mudança	é	lenta	e duradoura.			
	57	58	59	60					56	57	58	59			
13	Essa	chuva	não	ocorre	mais	todo	ano.	13	O armário	da casa	da minha	avó	é	antigo.	
	61	62	63	64	65	66	67		60	61	62	63	64	65	
14	A sombra	perto	do rio	é	muito	boa.		14	O estilete	é	uma	arma	perigosa.		
	68	69	70	71	72	73			66	67	68	69	70		
15	O telejornal	começa	às dez	da noite.				15	Ele	tem	a meta	de subir	o morro	de bicicleta.	
	74	75	76	77					71	72	73	74	75	76	
16	Meu	time	se consagrou	como	campeão	estadual.		16	O vão	da plataforma	é	estreito.			
	78	79	80	81	82	83			77	78	79	80			
17	A proposta	foi	inspecionada	pela	gerência.			17	Minha	tia	abriu	a caixa	de correspondência.		
	84	85	86	87	88				81	82	83	84	85		
18	O pássaro	canta	ao amanhecer.					18	O menu	inclui	um prato	muito	saboroso.		
	89	90	91						86	87	88	89	90		
19	A juventude	tinha	que revolucionar	a escola.				19	A cantora	terá	quatro	meses	de ensaio.		
	92	93	94	95					91	92	93	94	95		
20	Seu	limite	do cartão	foi	aumentado.			20	O time	continua	lutando	pelo	sucesso.		
	96	97	98	99	100				96	97	98	99	100		
Lista 9								Lista 10							
1	Esses	são	nossos	antigos	vizinhos.			1	Neste	caso,	dormirei	tranquilo.			
	1	2	3	4	5				1	2	3	4			
2	O inspetor	faz	a vistoria	completa.				2	Preciso	pegar	uma	caneta	amarela.		
	6	7	8	9					5	6	7	8	9		
3	A aula	dele	é	bastante	interessante.			3	Ele	dorme	num	leito	de palha.		
	10	11	12	13	14				10	11	12	13	14		
4	O congresso	volta	atrás	em sua	palavra.			4	Procurei	minha	amiga	em casa.			
	15	16	17	18	19				15	16	17	18			
5	O mau	tempo	finalmente	chegou	na serra.			5	O grêmio	ganhou	uma	quadra	de esportes.		
	20	21	22	23	24				19	20	21	22	23		
6	A explicação	pode	ser	encontrada	na tese.			6	A empresa	tem	uma	grande	produção	de metal.	
	25	26	27	28	29				24	25	26	27	28	29	
7	Os móveis	para	o quarto	chegarão	às três	da tarde.		7	Hoje	irei	precisar	de você.			
	30	31	32	33	34	35			30	31	32	33			
8	Durante	o dia	apague	a luz.				8	O dia	está	bom	para	passear	no parque.	
	36	37	38	39					34	35	36	37	38	39	
9	O clima	está	muito	seco	no interior.			9	Sem ele	o tempo	flui	num	ritmo	suave.	
	40	41	42	43	44				40	41	42	43	44	45	
10	Elas	traziam	o equipamento.					10	Ela	organizou	um grande	banquete.			
	45	46	47						46	47	48	49			
11	O trabalho	se tornou	cansativo	para	os alunos.			11	Ainda	não	se sabe	o dia	da prova.		
	48	49	50	51	52				50	51	52	53	54		
12	O pão	que eu	comprei	era	ótimo.			12	Meu	pai	gosta	de dormir	cedo	durante	a semana.
	53	54	55	56	57				55	56	57	58	59	60	61
13	Nossa	filha	foi	a primeira	a se formar	no exterior.		13	A paixão	dele	é	a natureza.			
	58	59	60	61	62	63			62	63	64	65			
14	Hoje,	eu	não	pude	fazer	minha	ginástica.	14	Eu	gosto	de tomar	banho	gelado	pela	manhã.
	64	65	66	67	68	69	70		66	67	68	69	70	71	72
15	A menina	estava	em cima	da escada.				15	Será	muito	difícil	conseguir	que eu	coma.	
	71	72	73	74					73	74	75	76	77	78	
16	O comércio	daqui	é	bem	tranquilo.			16	A intenção	é	ter	o apoio	do governador.		
	75	76	77	78	79				79	80	81	82	83		
17	Eu	tive	uma	prova	fácil	de geografia.		17	Desculpe,	mas	me atrasei	no casamento.			
	80	81	82	83	84	85			84	85	86	87			
18	O frio	deve	diminuir	ainda	este	ano.		18	O caminho	até	a fazenda	é	perigoso		
	86	87	88	89	90	91			88	89	90	91	92		
19	A médica	orientou	que eles	mudassem	o remédio.			19	A escuridão	do quarto	assutou	a criança.			
	92	93	94	95	96				93	94	95	96			
20	Nossas	atitudes	são	calmas.				20	O jogo	será	transmitido	à tarde.			
	97	98	99	100					97	98	99	100			

No estudo piloto realizado com três normo-ouvintes do sexo masculino e commédia de idade de 23,33 anos, a média do índice de reconhecimento das sentenças foi de 99,16%.

O primeiro participante do estudo piloto acertou 99,9% de todo o teste, tendo cometido apenas um erro na Lista 1. O segundo participante obteve 98,8% de acertos, tendo cometido nove erros na Lista 3, um erro na Lista 4, um erro na Lista 5 e um erro na Lista 9, totalizando 12 erros. Já o terceiro indivíduo acertou 99,3% do teste, tendo cometido seis erros na Lista 5 e um erro na Lista 7, sendo sete erros no total. Os erros apresentados pelos participantes incluíram falta de atenção na apresentação da sentença, levando a não repetição da mesma, ou trocas de palavras, ou ainda, trocas de palavras do plural para o singular e, em um caso, redução de 'pra' (usada na linguagem cotidiana) por 'para a'.

## DISCUSSÃO

Das 200 sentenças analisadas neste estudo, foram consideradas pelos juízes da primeira etapa do estudo 71 sentenças (35,5%) passíveis de serem modificadas. O coeficiente de confiabilidade interna foi alto tanto nas sentenças diferentes como nas comuns, mostrando que há concordância entre os juízes nos critérios avaliados. Esse dado é muito importante para uma seleção mais fidedigna do material de fala que será produzido.

A previsibilidade avaliada pelos juízes foi o critério que apontou com maior frequência para a necessidade de modificar as sentenças (Figura 1), sendo que a média das notas neste critério foi de 52,58% nas sentenças diferentes e de 56,76% nas sentenças em comum. Comparando a média das respostas nos critérios julgados pelos subgrupos dos juízes foi possível verificar que houve maior variação das respostas para o critério previsibilidade (Tabela 1 e 2).

Vale ressaltar que no critério previsibilidade quanto mais próximo de zero fosse a nota atribuída, menor seria a previsibilidade das sentenças. Na literatura, observa-se que este critério também foi utilizado na construção de outros materiais para avaliação da percepção da fala em adultos^(24)^normo-ouvintes e idosos com e sem perda auditiva^(16)^.

A presença de previsibilidade permite que a palavra-chave, normalmente localizada no final da sentença, seja previsível pelo contexto, em razão de que na sentença existe a presença de palavras semanticamente ligadas a ela. Caso haja baixa previsibilidade não é possível prever pelo contexto, não havendo outras palavras da sentença ligadas ao significado da palavra foco^(16)^. Dessa forma, quanto menos previsível for a sentença, mais fidedigno será a avaliaçãodo reconhecimento de fala.

Um estudo nacional^(16)^ com idosos, com e sem perda auditiva, utilizou sentenças de baixa e alta previsibilidade para avaliar o reconhecimento de fala no silêncio e ruído. Este estudo revelou que na condição de testagem no silêncio os idosos apresentam melhor desempenho que na condição ruído. Este estudo relata também que os idosos que apresentavam maiores graus de perdas auditivas, apontam mais benefício com o apoio do contexto. Os dados do estudo relatado mostram a importância de considerar o item previsibilidade na construção de testes de percepção de fala.

Da mesma forma, outro trabalho^(24)^ que foi realizado com indivíduos normo-ouvintes,quetinha o objetivo de avaliar o reconhecimento de fala, analisou a influência da previsibilidade das palavras utilizando sentenças com baixa e alta previsibilidade. O estudo constatou que as diferenças de pontuação entre os dois tipos de sentenças indicam o grau em que o ouvinte é capaz de se beneficiar com informações semânticas, sintáticas e prosódicas fornecidas pelo contexto da sentença, ou seja, o uso dessas sentenças indica até que ponto uma pessoa é capaz de fazer uso do contexto.

O grupo de reabilitadores foi o que julgou mais sentenças para serem modificadas no critério previsibilidade. Já o grupo de linguistas julgou mais necessário modificar a familiaridade e significado de sentenças. Acredita-se que os reabilitadores auditivos possivelmente por vivenciarem em suas rotinas clínicas a perspectiva do paciente deficiente auditivo, levem mais em conta o fator previsibilidade, uma vez que com o declínio das funções auditivas e alterações cognitivas, seus pacientes se esforçam para compreender a informação a ser memorizada, necessitam da inteligibilidade das palavras e se beneficiam do contexto linguístico da sentença como suporte^(25)^.

Em contrapartida, os linguistas analisam por uma visão lexical e consideram as palavras enquanto elementos isolados e, por esse motivo, o grau de familiaridade com cada palavra e seu significado são as particularidades mais consideradas para seleção e controle dos materiais por esse grupo^(26)^. Visto isso, percebe-se a importância da abordagem desses dois grupos com perspectivas e olhares diferentes para uma atualização mais efetiva da lista de sentenças.

A familiaridade é a frequência com a qual se escuta determinado *input* linguístico e com a qual esse *input* é efetivamente usado, ou seja, o quanto é conhecida a expressão^(27)^. Verificou-se que este critério também tem sido utilizado na construção de materiais de fala utilizados em adultos^(6)^ e crianças normo-ouvintes^(4,5)^, adultos usuários de IC^(11)^, idosos com e sem perda auditiva^(16)^ e em crianças, adolescentes e adultos falantes nativos de PB^(28)^.

O critério de familiaridade é essencial na construção de testes de percepção de fala, principalmente os direcionados para crianças, já que a utilização de palavras desconhecidas pela criança pode gerar resultados errôneos em relação ao reconhecimento auditivo dos sons da fala.

Um estudo^(5)^ que tinha como objetivo aplicar um material de percepção de fala em conjunto fechado para analisar o índice percentual de reconhecimento de fala (IPRF) em crianças com deficiência auditiva constatou que, tão importante quanto o estímulo de fala escolhido, são os critérios de familiaridade e o significado das palavras.

A quantidade de sentenças que precisam ser modificadas a partir da análise dos juízes da primeira etapa (35,5%) para tornar este banco de fala mais fidedigno e aplicável na prática clínica mostrou-se reduzido. Esse resultado mostra que este banco de fala, já revisado em 1998, apresenta um conteúdo que, além de balanceado foneticamente, é familiar, com significado e baixa previsibilidade.

Um critério que auxiliou na atualização das sentenças foi a experiência dos juízes. Foi possível verificar que tanto os linguistas como os reabilitadores tinham, em sua maioria, pós-graduação na área de interesse na presente pesquisa (84,21%). Os reabilitadores tinham uma média de 17 anos de experiência na área de reabilitação auditiva e com experiência na aplicação de testes de percepção de fala. Além disso, os juízes linguistas tinham mais de uma área de atuação, sendo a maioria nas áreas de fonética e fonologia - áreas de grande importância para estudos sobre a produção e a percepção dos sons da fala^(29)^.

A maioria dos juízes reabilitadores deste estudo utilizam os testes de reconhecimento de fala na sua prática clínica (75%). Um estudo^(13)^ descritivo realizado sobre esses testes de fala utilizados nos centros de implante coclear no Brasil verificou que 63% dos serviços avaliados aplicam os testes em sala de terapia e que não há uniformidade nas condutas de avaliação com o uso de testes de percepção de fala.

Além das 71 sentenças que foram apontadas pelos juízes para serem modificadas na primeira etapa do estudo, outras 28 sentenças foram julgadas para serem modificadas pelos juízes da segunda etapa. Considerando as modificações da primeira (71 sentenças) e segunda etapa (28 sentenças) foram modificadas ao total 99 sentenças (49,50%) da lista original com intuito de elaborar uma lista de sentenças direcionada para a avaliação da percepção da fala de sujeitos e adequada para aplicação clínica.

Na segunda etapa, o elemento que mais foi identificado e modificado nas sentenças foi nome próprio. Acredita-se que a literatura recomende a exclusão de nomes próprios nas sentenças para evitar regionalismos, uma vez que alguns nomes podem não ser familiares e habituais e impossibilitem tradução para outros idiomas.

Outro critério modificado adotado no presente estudo foi analisar se as sentenças não apresentavam mais de sete vocábulos fonológicos. Controlar o número de palavras já vem sendo recomendado desde 1955^(22)^ para evitar que o acesso a memória interfira na avaliação do reconhecimento de fala. Nesse estudo o autor recomenda que as sentenças não tenham mais do que 12 palavras.Já em um estudo de 1979^(23)^ foi recomendado que as sentenças não ultrapassassem sete sílabas. Um trabalho nacional^(7)^ que utilizou os critérios de ambos os estudos citados considerou então que as sentenças deveriam apresentar de quatro a sete vocábulos fonológicos.

Um estudo relata que na avaliação da memória de trabalho em pessoas com perda auditiva a alça fonológica é acessada. Nos indivíduos com prejuízo nas funções sensoriais auditivas pode haver problemas para acessar a alça fonológica e processar a informação auditiva. Para compensar essa diminuição da percepção, pacientes com IC dependem mais do processamento *top-down* que faz uso do acesso fonológico/ lexical e do armazenamento da memória de longo prazo^(30)^.

Dessa forma, o controle de palavras é muito importante nas sentenças para evitar esforço cognitivo nestes indivíduos, o que poderia levá-los a cometerem erros em testes de reconhecimento de fala.

Além do controle de vocábulos fonológicos por sentença foi realizado o controle de quantidade de vocábulos fonológicos por lista. Observou-se que em outro teste muito utilizado para avaliar a porcentagem de reconhecimento de fala^(7)^, especialmente de usuários de implante coclear, as listas são constituídas por 50 vocábulos fonológicos. No presente estudo optou-se por 100 vocábulos fonológicos por lista para que as mesmas sejam utilizadas tanto para o cálculo do índice percentual de reconhecimento de fala, como também do limiar de reconhecimento de fala. Por esse mesmo motivo neste estudo foi decidido deixar maior quantidade de sentenças por lista do que o estudo original que apresentava apenas 10 sentenças por lista^(20)^. Na literatura verificou-se que outros testes mais recentes de reconhecimento de fala, como o HINT^(8)^, apresentam a mesma quantidade de sentenças por lista que o presente estudo.

No estudo piloto realizado para avaliar a aplicabilidade clínica das listas constatou-se que os participantes obtiveram escores muito próximos a 100% de acertos. Nenhum dos participantes relatou dificuldades na realização do teste. Acredita-se que os indivíduos tiveram excelente reconhecimento de fala pois além das listas apresentarem conteúdo com bom significado e familiaridade, houve o cuidado do controle do número de vocábulos fonológicos por sentença para evitar questões atencionais e/ou de memória, conforme vem sendo recomendado pela literatura^(7,22,23,30)^.

A próxima etapa deste trabalho será a gravação das sentenças em estúdio e uso das sentenças gravadas em ensaios com sujeitos normo-ouvintes e indivíduos com diferentes graus de perdas auditivas visando à padronização do limiar de reconhecimento de fala no silêncio e no ruído. Para tanto as sentenças gravadas serão inseridas no software perSONA, desenvolvido pelo Laboratório de Vibrações e Acústica da UFSC que permite avaliar a percepção de fala com ruído competitivo, podendo reproduzir cenas acústicas complexas estáticas e dinâmicas.

Este estudo foi de grande importância, pois mostra a relevância de atualizar os testes de reconhecimento de fala para o cotidiano e contribuir para a criação de um material fidedigno para a prática clínica que possa auxiliar na padronização da avaliação de percepção de fala no Brasil com normo-ouvintes e com indivíduos com diferentes graus de perda auditiva.

## CONCLUSÃO

Após análise dos dados e resultados obtidos, considera-se que o presente estudo alcançou o seu objetivo, ou seja, adaptar uma lista de sentenças para avaliar o reconhecimento de fala para adultos falantes de PB.

Foi possível atualizar as sentenças do banco de fala, sendo o critério de previsibilidade o mais apontado pelos juízes na primeira fase do estudo. Já na segunda etapa, foi a exclusão de nomes próprios e sentenças que não continham baixo nível de abstração e boa semântica.
